# Seroprevalence and risk factors of *Toxoplasma gondii* in urban cats from China

**DOI:** 10.1186/s12917-022-03427-w

**Published:** 2022-09-01

**Authors:** Ningbo Xia, Nuo Ji, Longjiao Li, Yuan Huang, Congshan Yang, Xuefang Guo, Qinghong Guo, Bang Shen, Lihua Xiao, Yaoyu Feng

**Affiliations:** 1 Guangdong Laboratory for Lingnan Modern Agriculture, Center for Emerging and Zoonotic Diseases, College of Veterinary Medicine, South China Agricultural University, Guangzhou, 510642 China; 2 Chongqing Three Gorges Vocational College, Chongqing, 404155 China; 3 State Key Laboratory of Agricultural Microbiology, Huazhong Agricultural University, 430070 Wuhan, China; 4 College of Animal Science and Technology, Anhui Agricultural University, Hefei, 230036 China

**Keywords:** Toxoplasmosis, Epidemiology, Serology, Definitive host, Cat

## Abstract

**Background:**

*Toxoplasma gondii* infects almost all warm-blooded animals, and cats play a crucial role in the epidemiology of *T. gondii* as the definitive host. Despite sporadic reports on the seroprevalence of *T. gondii* in domestic cats, systematic surveys are lacking and some regions remain in China uninvestigated.

**Methods:**

A total of 1,521 serum samples were collected from 10 regions of China and analyzed by antibodies against *T. gondii* by ELISA with the purpose of identifying risk factors of *T. gondii* infection in cats across China and obtaining seroprevalence data from some previously uninvestigated areas.

**Results:**

Antibodies to *T. gondii* were detected in 62 of 1,478 (4.2%) urban pet cats and in 9 of 43 (20.9%) stray cats. Among the regions examined, the prevalence was 13% in Sichuan, 12.8% in Chongqing, 6.4% in Hunan, 2.5% in Hubei and 0.9% in Guangdong. Additionally, this is the first report on the seroprevalence of *T. gondii* in urban pet cats from Qinghai (6.2%), Anhui (3.1%), Jiangxi (2.5%), Shaanxi (2.4%) and Ningxia (1.6%). The age and lifestyle (stray or pet) of cats were identified as the risk factors for seropositivity by multivariate analysis of the data.

**Conclusions:**

Our findings improve our understanding of seroprevalence and risk factors of *T. gondii* infection in cats across China, and provide useful information for the formulating of preventive and control measures against this widespread zoonotic parasite.

**Supplementary Information:**

The online version contains supplementary material available at 10.1186/s12917-022-03427-w.

## Introduction

*Toxoplasma gondii*, an obligate intracellular protozoan parasite with cats as the definitive host and warm-blooded animals as intermediate hosts, infects one-third of the world population and numerous animals, causing toxoplasmosis [1]. *T. gondii* has a complex life cycle, including the asexual cycle in intermediate hosts (such as humans, pigs, and sheep) and the sexual development restricted to felines [2, 3]. In the intestinal epithelium of cats, *T. gondii* differentiates into female and male gametocytes, allowing sexual reproduction and generation of environmentally resistant oocysts [1–3]. There are two primary routes for the intermediate host to acquire *T. gondii* infection: eating undercooked meat containing tissue cysts [1–3], and ingesting food or water contaminated by oocysts [1–3]. *T. gondii* infection can cause abortion, stillbirth, infants with hydrocephalus, and death of organ transplant recipients and immunodeficient patients [4]. 

Cats can excrete millions of oocysts and play a vital role in the epidemiology of *T. gondii* [1]. In recent years, with the improvement of living standards, the number of cats raised in China has been on a steady rise [5]. Whether the risk of toxoplasmosis in cats would increase remains unknown. The seropositivity of *T. gondii* antibodies in cats, although not an indicator for the current shedding of the parasite, is a useful indicator of infection in a cat population and can be used to evaluate the infection risk in both the definitive and intermediate hosts in the same area [6]. The seroprevalence for *T. gondii* in cats was reported to vary between 30% and 40% worldwide [5, 7]. Although previous studies have reported the prevalence of toxoplasmosis in cats in some areas of China [8, 9], related information is still lacking in many provinces. In addition, seroprevalence studies are needed to identify the risk factors associated with the occurrence of *T. gondii* infections in cats.

In this study, we examined the prevalence of toxoplasmosis in cats in 10 provinces of China, including five previously uninvestigated provinces. Our study aimed to determine the seroprevalence and risk factors of *T. gondii* in urban cats from China. The results provide useful information to parasitologists, veterinarians, biologists, and public health workers in the prevention and control of this common zoonotic parasite.

## Materials and methods

### Study area and sampling

**Fig. 1:**
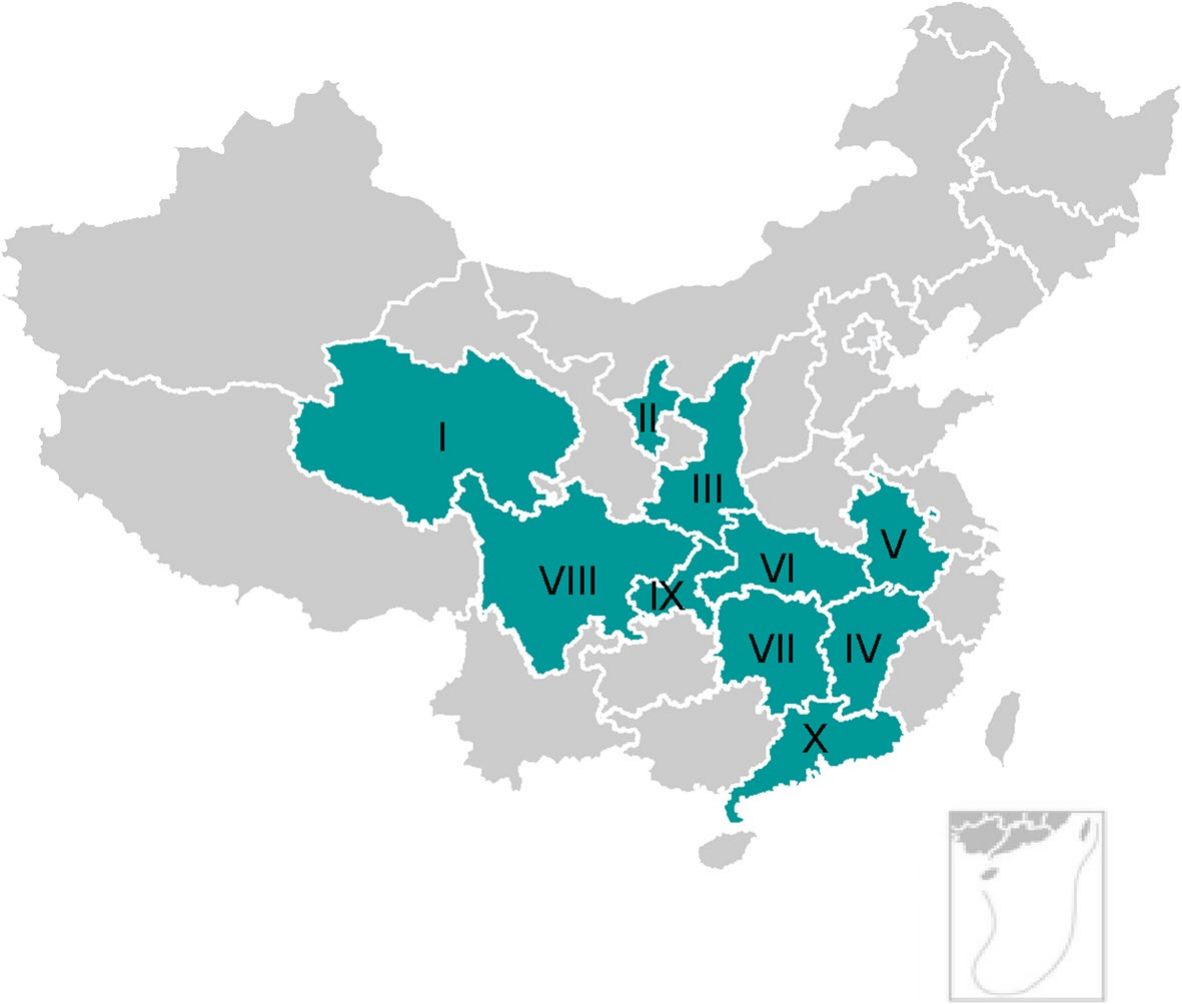
Location of sampling for the study of seroprevalence of *Toxoplasma gondii* infection in cats in China. I, Qinghai; II, Ningxia; III, Shaanxi; IV, Jiangxi; V, Anhui; VI, Hubei; VII, Hunan; VIII, Sichuan; IX, Chongqing; X, Guangdong

A total of 1,521 serum samples were collected from 10 provinces and regions of China from 2021 to 2022 (Fig. 1 and Table S1). Information on the sex, age, breed, food, water source, physical condition and lifestyle (stray or pet) was obtained from the pet owners or at the time of sample collection. The serum samples were stored at − 20 °C until further analysis.

### Serological examination

The serum samples were analyzed using the commercial kit ID Screen® Toxoplasmosis Indirect Multi-species ELISA (ID.vet, Grabels, France), which detects anti-*Toxoplasma* IgG antibodies. Briefly, each serum sample (10 µl) was diluted with the buffer (90 µl) at a ratio of 1:9 in a test well and incubated at 21 °C for 45 min. After washing, the microplates were incubated with the secondary antibody at 21 °C for 30 min, followed by reaction with the substrate solution (TMB) for 15 min. The absorbance value at 450 nm was measured with a microplate reader (BioTek Instruments Inc., Winooski, VT, USA). The S/P value was calculated for each sample by the following equation: S/P% = [(OD_S_-OD_NC_) /(OD_PC_-OD_NC_)] × 100, where OD_S_ is the optical density (OD) of the sample; OD_PC,_ the OD of the positive control; OD_NC._ the OD of the negative control. S/P% ≥ 50% was regarded as a positive sample, while S/P% ≤ 40% as a negative sample. Samples judged as doubtful were re-tested, with a second doubtful result being treated as a negative sample.

### Statistical analysis

The seroprevalence data were initially analyzed using chi-square implemented in SPSS 20.0 (IBM Inc., Chicago, IL, USA). The odds ratio (OR) value was calculated with a 95% confidence interval. Variables with a *P* value ≤ 0.2 in a univariate analysis of potential risk factors were further analyzed using the multivariate logistic regression analysis. A *P* value of < 0.05 was considered significant.

## Results and discussion

*Toxoplasma gondii*, a widespread zoonotic pathogen, was estimated to infect one-third of the world population and all domesticated animals [1]. Cats are definitive hosts for *T. gondii* and can shed a large number of oocysts. Therefore, cats play a crucial role in the transmission of *T. gondii*, and data on the seroprevalence of *T. gondii* in cats are necessary for the development of effective prevention and control measures against toxoplasmosis. In this study, we found that the overall seroprevalence of *T. gondii* in urban pet cats and stray cats in China was 4.2% and 20.9%, respectively.

Although some reports on the prevalence of *T. gondii* antibodies in domestic cats have been published, there is still a paucity of data in China. Several previous studies have examined the prevalence of *T. gondii* antibodies in domestic cats from China, reporting seroprevalence rates of 20.8%, 23.7% and 57.3% in domestic cats in Liaoning, Guangdong and Hebei, respectively [10–12]. In addition, antibodies to *T. gondii* have also been detected in domestic cats from Beijing [13–17], Inner Mongolia [18], Gansu [19–21], Shandong [22, 23], Jiangsu [24, 25], Shanghai [26, 27], Zhejiang [28], Fujian [29, 30], Henan [31–34], Hubei [35], Hunan [36], Chongqing [37], Sichuan [38], Guangxi [39] and Hainan [40]. In the present study, samples were taken from 5 regions where *T. gondii* infection in domestic cats has have never reported, and the positive rate was found to be 3.1% (6 of 192) in Anhui, 2.5% (2 of 81) in Jiangxi, 6.2% (5 of 81) in Qinghai, 2.4% (1 of 41) in Shaanxi and 1.6% (4 of 244) in Ningxia (Table 1). On the other hand, despite previous reports of the seroprevalence rates of 31.4%, 28.0% and 18.0% for domestic cats in Hubei, Hunan and Guangdong, respectively [35, 36, 41], no recent information is available from these areas. In the present study, the seroprevalence in Hubei (2.5%), Hunan (6.4%) and Guangdong (0.9%) was much lower than the previously reported rates (Table 1).

**Table 1:** Prevalence of *Toxoplasma gondii* antibodies in domestic cats

Region	Province/City	Test time	No. tested	No. positive	% Positive
Northwest China	Qinghai	2021–2022	81	5	6.2%
	Ningxia	2021	244	4	1.6%
	Shaanxi	2021	41	1	2.4%
Eastern China	Jiangxi	2021–2022	81	2	2.5%
	Anhui	2021	192	6	3.1%
Central China	Hubei	2021	406	10	2.5%
	Hunan	2021	141	9	6.4%
Southwest China	Sichuan	2021–2022	100	13	13%
	Chongqing	2021	86	11	12.8%
Southern China	Guangdong	2021–2022	106	1	0.9%
Total			1,478	62	4.2%

The prevalence of *T. gondii* among domestic cats across China in the present study is lower than the values reported in some previous studies [35, 36, 41]. There are at least two possible explanations for the lower values in this study. Firstly, the public awareness of *T. gondii* prevention in cats has gradually increased, encouraging pet owners to pay more attention to scientific breeding and environmental sanitation. Secondly, this study has mainly focused on samples from urban regions, with almost no samples collected from rural areas, where the seroprevalence is known to be higher than in urban areas. Nevertheless, despite the low prevalence rate, some cats with no outdoor activities are infected. One explanation is that people carry *T. gondii* oocysts into the home through shoe soles or objects contaminated with oocysts, causing the infection of cats with *T. gondii*. It is also possible that owners fed their cats with undercooked meat containing *T. gondii* cysts.

Common potential risk factors for the infection of cats with *T. gondii* include sex, age, food, and water source. Different from other studies, the present study has identified no significant association between sex, diet, or water source and *T. gondii* positivity (Table 2). Nevertheless, the seropositivity was found to increase with the age of cats (Tables 2 and 3), which may be related to increased exposure to *T. gondii* through food, water and outdoor activities as the cat grows. In addition, *T. gondii* antibodies were detected (3 out of 124 samples) in cats of ≤ 3 months, indicating the likely occurrence transplacental infection or maternally transferred antibodies in some young domestic cats. Moreover, the seroprevalence of *T. gondii* was found to be related to the lifestyle of cats, with a higher positive rate in stray cats (20.9%) than in household cats (4.2%) (Tables 2 and 3). This is not surprising, as stray cats have a wider roaming range than household cats and are more likely to be exposed to *T. gondii* oocysts. In addition, stray cats could also become infected by hunting rodents. It is also noteworthy that a high positive rate in stray cats indicates the possible presence of large numbers of *T. gondii* oocysts in the environment, increasing the risk of infection in other animals and humans.

**Table 2:** Analysis of potential risk factors in seroprevalence of *Toxoplasma gondii* in domestic cats (1478) in 10 regions in China

Variable	N (%)	OR ^c^	95% CI	χ^2^	*P* value
**Sex**					
Female	606 (3.8)	0.976	0.554–1.721	0.000	1.000
Male	695 (3.9)				
Unknown	177 (6.8)				
**Age(years)**					
≤ 1	566 (2.3)	0.300	0.152–0.592	12.176	0.000^d^
1 < Age ≤ 2	183 (6.0)	1.629	0.795–3.336	1.299	0.254
> 2	175 (8.6)	2.832	1.453–5.520	8.824	0.003^d^
Unknown	554 (4.2)				
**Food**					
Commercial cat food	1134 (3.7)	0.641	0.266–1.545	0.541	0.462
Homemade cooked food	5 (0)	NA	NA	0.000	1.000
Animal organs	29 (3.4)	0.884	0.118–6.640	0.000	1.000
Other^a^	72 (6.9)	1.952	0.749–5.091	1.163	0.281
Unknown	238 (5.9)				
**Water**					
Tap water	598 (4.7)	1.445	0.798–2.618	1.149	0.284
Filtered water	529 (3.0)	0.620	0.335–1.146	1.930	0.165^d^
Other^b^	49 (6.1)	1.605	0.481–5.363	0.163	0.687
Unknown	302 (5.0)				
**Lifestyle**					
Domestic cats	1478 (4.2)	0.165	0.076–0.360	22.671	0.000^d^
Stray cats	43 (20.9)				

Note: Some cats eat/drink two or more types of food/water

^a^Such as snacks, canned, freeze-dried, lean meat or some cats eat two or more types of food

^b^Such as plain water, mineral water or mix of two or more types of water

^c^Odds ratio

^d^Selected for multivariate analysis

**Table 3:** Risk factors for *Toxoplasma gondii* infection in cats in China identified by the multivariate logistic regression analysis

Variable	Multiple logistic regression					
	Coefficient	Standard error	Wald	Degrees Of freedom	*p*-value	OR (95% CI)
Age	0.017	0.006	8.287	1	0.004	1.017 (1.005–1.029)
Water	0.601	1.041	0.333	2	0.564	1.824 (0.237–14.036)
Lifestyle	-1.799	0.397	20.574	1	0.000	0.165 (0.076–0.360)

## Conclusions

In this study, the seroprevalence of *T. gondii* was found to be 4.2% in urban pet cats across China. Despite a lower positive rate than in previous studies, we should still pay attention to the risk of human toxoplasmosis through ingesting *T. gondii* oocysts shed by pet and stray cats. While further investigations should be conducted on the prevalence of *T. gondii* in cats of rural regions, data from the study enriches our understanding of transmission and risk factors of *T. gondii* in cats in China in recent years, and provides the much-needed data for the formulation of preventive and control measures against toxoplasmosis.

## Supplementary Information

**Additional file 1:** **Table S1.** Information for sampling locations for the study of seroprevalence of *Toxoplasma gondii* infection in cats in China.

## Data Availability

The datasets used and/or analyzed during the current study are available from the corresponding author on reasonable request.

## Abbreviations

ELISA: Enzyme-linked immunosorbent assay
IFAT: Indirect fluorescence antibody test
IHA: Indirect hemagglutination assay
LAT: Latex agglutination test
MAT: Modified latex agglutination test
