# Supplementary material for: Seroprevalence and risk factors of Toxoplasma gondii in urban cats from China
Source: BMC Vet Res. 2022 Sep 1;18:331. doi: 10.1186/s12917-022-03427-w (PMC9434894; doi:10.1186/s12917-022-03427-w)
Supplement: Supplementary file 1 — Additional file 1: Table S1. Information for sampling locations for the study of seroprevalence of Toxoplasma gondii infection in cats in China. [file 12917_2022_3427_MOESM1_ESM.docx]

**Supporting information**

**Table S1. Information for sampling locations for the study of seroprevalence of *Toxoplasma gondii* infection in cats in China**

| Region | Location | Region | City | Longitude and latitude |
| --- | --- | --- | --- | --- |
| Northwest China | I | Qinghai | Xining | 101.74 °E 36.56°N |
|  | II | Ningxia | Yinchuan | 106.27°E 38.47°N |
|  | III | Shaanxi | Ankang | 109.02°E 32.7°N |
| Eastern China | IV | Jiangxi | Pingxiang | 113.85°E 27.6°N |
|  |  |  | Nanchang | 115.89°E 28.68°N |
|  | V | Anhui | Hefei | 117.27°E 31.86°N |
| Central China | VI | Hubei | Jingzhou | 112.23°E 30.33°N |
|  |  |  | Suizhou | 113.37°E 31.72°N |
|  |  |  | Xiaogan | 113.91°E 31.92°N |
|  |  |  | Wuhan | 114.31°E 30.52°N |
|  |  |  | Xianning | 114.32°E 29.85°N |
|  |  |  | Ezhou | 114.88°E 30.40°N |
|  | VII | Hunan | Changsha | 113.00°E 28.21°N |
| Southwest China | VIII | Sichuan | Yaan | 103.00°E 29.98°N |
|  |  |  | Chengdu | 104.07°E 30.67°N |
|  | IX | Chongqing | Wanzhou | 108.40°E 30.82°N |
| Southern China | X | Guangdong | Maoming | 110.88°E 21.68°N |
|  |  |  | Guangzhou | 113.23°E 23.16°N |
